# GenPop-An Online Tool to Analyze Human Population Genetic Data

**DOI:** 10.6026/97320630016149

**Published:** 2020-02-29

**Authors:** B Arundhati Mahesh, E Kannan, G Dicky John Davis, P Venkatesan, PK Ragunath

**Affiliations:** 1 Department of Bioinformatics, Sri Ramachandra Institute of Higher Education and Research (DU), Porur, Chennai ,Tamilnadu, India- 600116; 2 VelTech Rangarajan Dr. Saguthala R&D Institute of Science and Technology, Avadi, Chennai ,Tamilnadu, India - 600062

**Keywords:** Geneticist, epidemiologist, Hardy-Weinberg equilibrium, PHP

## Abstract

GenPop is a web based online cross platform tool developed to help Geneticist and Epidemiologist to deal with association studies in analyzing human population genetic data. The tool features include descriptive analysis such as Hardy-Weinberg equilibrium test, chi-square p-value and analysis of single nucleotide polymorphisms (SNPs) with multiple inheritance models such as dominant, recessive, allelic, genotype, odd's ratio and relative risk at 95% confidence interval and analysis of multiple SNPs including haplotype frequencies and linkage disequilibrium for a pair of biallelic markers. This is a user-driven human population genetic data analysis tool that is easily scalable and acceptable with multiple implementations of different algorithms. GenPop has been developed using PHP, JavaScript and with PHPExcel library to analyse the genetic data for case control studies.

## Background

Genetic epidemiology deals with the study the role of genetic factors involved in determining health and diseases in families and as well in populations which seeks to derive statistical and quantitative analysis of how genetics work in larger groups. There are various software packages developed for analyzing human genetic data which rely on computer-based algorithm that is not passable in certain instances, few packages provide a single function and are difficult to install and use. Statistical packages can be used to perform these study analysis, but an assistance of computational tool is mainly needed by researcher to perform specific analysis like HWE, haplotype estimation, and at times difficulty in integrating results from different packages at a shorter time span [1]. Thereby as a constraint of trend in the use of worldwide Web technology and Web design that aims at enhancing creativity, information sharing and communication among users in analysing the case control data.

## Materials and Methods:

**Figure 1:**
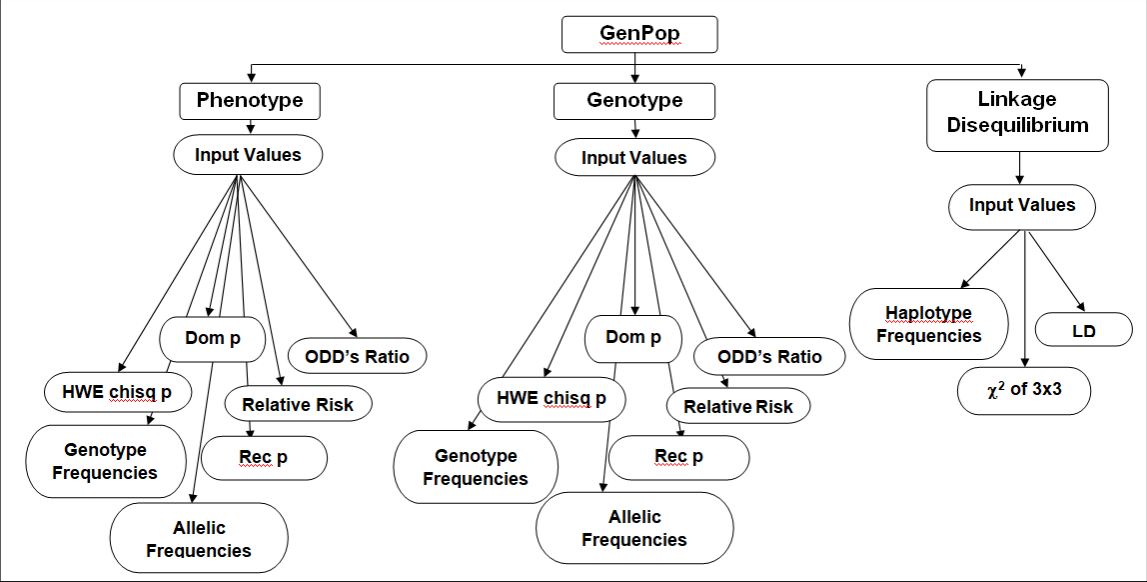
Flowchart for GenPop Tool

GenPop is developed using JavaScript, which is a dynamic, integrated, and prototype-based language that makes it easy to use and flexible [2]. PHP is a server-side scripting language with PHPExcel library to analyse. The example used in help menu is taken from elsewhere [3]. The workflow of the tool is shown in Figure 1 flowchart.

## Results

### Descriptive and association analysis of SNPs:

**Figure 2:**
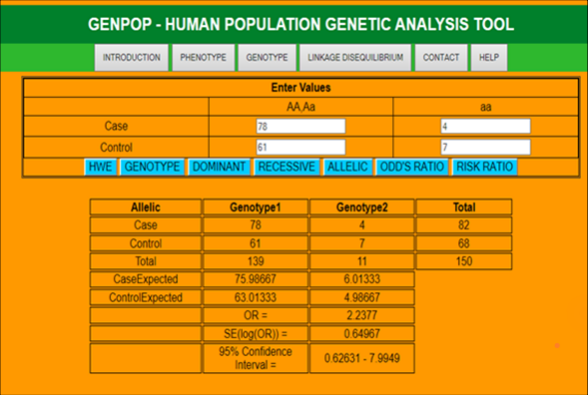
ODD's Ratio, Standard Error and 95% confidence interval for the observed Phenotype

**Figure 3:**
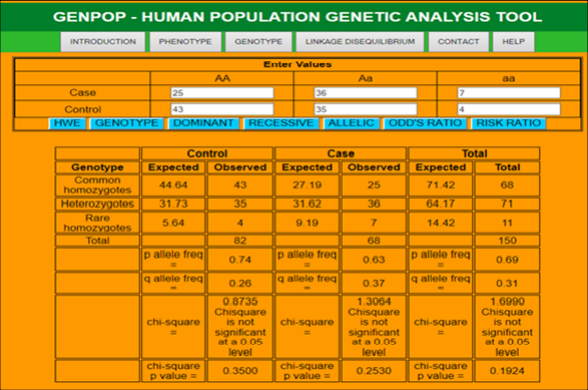
P and q allele frequency with chi-square p value for the observed Genotype

Testing Hardy-Weinberg equilibrium is commonly performed for analyzing genetic marker data such as SNPs in population studies. The chi-square test determines if a sample data matches a population. The p and q allelic frequencies for the observed phenotype or genotype are calculated to get chi-square p value (Figure 3). The tool also provides ODD's ratio and Risk Ratio with 95% confidence interval for phenotype or genotype using logistic regression analysis (Figure 2).

### Linkage disequilibrium analysis:

**Figure 4:**
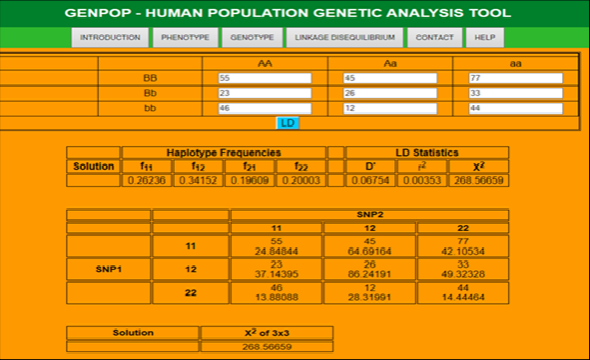
Haplotype Frequencies, LD Statistics, χ^2^ of 3x3

Linkage disequilibrium (LD) refers to the dependence of alleles from neighboring loci and can provide information on population histories and disease mapping. A widely used statistic measuring pairwise LD between single nucleotide polymorphisms (SNPs) and or multi allelic markers is Hedrick's D',r^2^ and χ^2^ which is based on two-locus haplotype frequencies [4] as shown in Figure 4.

## Discussion:

There are many computer programs in population genetics that have been successful in hiding the complexity of the computations from the user but they often rely on assumptions that are crucial for a correct interpretation of the results [5]. The research community uses the R statistical and computing language since all R code is open source.The language allows functions to be evaluated and modified by the user [6].GenPop is a tool developed that gives integrated results for a single input based on the user's choice without much time consumption and is free and easily available on web as an online tool.

## Conclusions:

GenPop is an online cross platform tool that is useful in performing analysis of association studies based on single nucleotide polymorphisms (SNPs) or biallelic markers.
